# Determination of Olanzapine and *N*-desmethyl-olanzapine in Plasma Using a Reversed-Phase HPLC Coupled with Coulochemical Detection: Correlation of Olanzapine or *N*-desmethyl-olanzapine Concentration with Metabolic Parameters

**DOI:** 10.1371/journal.pone.0065719

**Published:** 2013-05-31

**Authors:** Mong-Liang Lu, Chia-Hui Lin, Yi-Chuan Chen, Huai-Chih Yang, Tzu-Hua Wu

**Affiliations:** 1 Department of Psychiatry, Taipei Medical University-Wan Fang Medical Center, Taipei, Taiwan; 2 Department of Psychiatry, College of Medicine, Taipei Medical University, Taipei, Taiwan; 3 Department of Clinical Pharmacy, School of Pharmacy, College of Pharmacy, Taipei Medical University, Taipei, Taiwan; Chiba University Center for Forensic Mental Health, Japan

## Abstract

**Background:**

Olanzapine (OLZ) is one of the most prescribed atypical antipsychotic drugs but its use is associated with unfavorable metabolic abnormalities. N-desmethyl-olanzapine (DMO), one of the OLZ metabolites by CYP1A2, has been reported to have a normalizing action on metabolic abnormalities, but this remains unclear. Our aim was to explore the correlation between the concentrations of OLZ or DMO with various metabolic parameters in schizophrenic patients.

**Methods:**

The chromatographic analysis was carried out with a solvent delivery system coupled to a Coulochem III coulometric detector to determine OLZ and DMO simultaneously in OLZ-treated patients. The correlation between the concentration of OLZ or DMO and the metabolic parameters was analyzed by the Spearman rank order correlation method (*r*_s_).

**Principal Findings:**

The established analytical method met proper standards for accuracy and reliability and the lower limitation of quantification for each injection of DMO or OLZ was 0.02 ng. The method was successfully used for the analysis of samples from nonsmoking patients (n = 48) treated with OLZ in the dosage range of 5–20 mg per day. There was no correlation between OLZ concentrations and tested metabolic parameters. DMO concentrations were negatively correlated with glucose (*r*_s_ = –0.45) and DMO concentrations normalized by doses were also negatively correlated with insulin levels (*r*_s_ = –0.39); however, there was a marginally positive correlation between DMO and homocysteine levels (*r*_s_ = +0.38).

**Conclusions:**

The observed negative correlations between levels of DMO and glucose or insulin suggest a metabolic normalization role for DMO regardless of its positive correlation with a known cardiovascular risk factor, homocysteine. Additional studies of the mechanisms underlying DMO’s metabolic effects are warranted.

## Introduction

Olanzapine (LY170053; 2-methyl-4-(4-methyl-1-piperazinyl)-10H- thieno[2,3b][1], [5]benzodiazepine; OLZ) is a second-generation antipsychotic drug. The U.S. Food and Drug Administration (FDA) has approved OLZ for treatment of schizophrenia, acute treatment of manic or mixed episodes associated with bipolar I disorder, and maintenance treatment of bipolar I disorder. OLZ is marketed as a neuroleptic with a low degree of extrapyramidal side effects [1], [2], although a recent study reported that patients treated with OLZ tended to develop various metabolic abnormalities when compared to the other atypical antipsychotics [3].

Following oral administration, 57% of OLZ is excreted as its metabolites. OLZ is mainly metabolized to 10-N-glucuronide, 4'-N-desmethylolanzapine (DMO), olanzapine-N-oxide through uridine diphosphate glucuronosyltransferase (UGT) 1A4, cytochrome P450 (CYP) 1A2 isoenzymes and a flavin-containing monoxygenase, respectively [4]. The influences of gender, smoking habits, genetic variants, and certain drug interactions on concentrations of OLZ or DMO normalized by doses must be considered in order to provide optimal dosage of OLZ for disease management [5]. Various smoking behaviors may influence the OLZ concentration and pharmacokinetic parameters [6], [7] due to the increased activity of CYP1A2 associated with smoking. Even though it is known that 10-N-glucuronide is the most abundant metabolite and formation of DMO was reported to be correlated with the clearance of OLZ [4], it has been suggested that the metabolite DMO, but not OLZ itself, has a normalizing effect on metabolic abnormalities [8]. In another study with children and adolescents, OLZ concentration was significantly correlated with DMO (r = 0.567; P<0.0005) [9].

Metabolic abnormalities induced by OLZ [10] include weight gain [11], hyperglycaemia, dyslipidaemia [12], and hyperprolactinemia [3]. Preliminary evidence suggests a dose-response relationship between OLZ plasma concentrations and metabolic outcomes [13]; however, only a few studies have investigated a possible association between plasma concentrations of OLZ metabolites and metabolic outcomes in a limited number of subjects (n = 10∼16). For example: weight change correlated inversely with the plasma concentration level of DMO [8] and levels of the other metabolic parameters such as insulin correlated positively with the ratio of OLZ to DMO concentration [14] in OLZ-treated patients. An earlier study also revealed a strong association between metabolic syndrome and hyperhomocysteinemic patients with bipolar disorder and schizophrenia treated with second generation antipsychotics [15]. In order to clarify the role of DMO in OLZ-related metabolic changes, the steady-state trough concentrations of OLZ and its metabolite DMO were determined by a validated high performance liquid chromatography with electrochemical detection (HPLC-ECD) system which analyzed OLZ and DMO simultaneously in non-smoking patients with schizophrenia or schizoaffective disorder and who were treated with oral OLZ as the only antipsychotic drug. The correlations of concentration/dose (C/D) ratios of OLZ or DMO levels with patients’ metabolic parameters were analyzed.

## Materials and Methods

### Subjects and ethics statement

Forty-eight schizophrenic inpatients or outpatients (30 females and 18 males), aged 21 to 62, were recruited for this drug monitoring study. The decision to request therapeutic drug monitoring was made by the patients’ psychiatrists on the basis of clinical considerations. All patients were stable with at least three months of OLZ therapy during the period June 2007-Oct. 2008. This study was approved by the institutional review board and the ethics committee of the Taipei Medical University. A clinician who was experienced in the evaluation of mental illness assessed by a direct examination of participants, their understanding of all the procedures and capacity to consent [16]. The participants were included in the study only if they had the full capacity to consent. After a psychiatrist explained the study procedures and possible adverse events, the patients gave written informed consent to participate in the study. Participants’ personal identification features were removed and case information is for research work only. All potential participants who declined to participate or otherwise did not participate were eligible for treatment and were not disadvantaged in any other way by not participating in the study. Samples were drawn in the morning, approximately 12 hours after the last dose of OLZ. The blood samples were either subjected to biochemical analysis (glucose, insulin, homocysteine, c-peptide, prolactin, cholesterol, and triglyceride) or immediately centrifuged for 15 minutes at 4°C at 2000 g, and the plasma was separated and stored at –20 degree C until analysis for determinations of OLZ and DMO.

### Chemicals

OLZ was kindly donated by Eli Lilly (Indianapolis, IN, USA). DMO was purchased from BDG synthesis (Wellington, New Zealand). Clozapine, 8-chloro-11-(4-methyl-1-piperazinyl)-5H-dibenzo[b,e][1], [4]diazepine, used as an internal standard (IS), was purchased from Sigma-Aldrich (Saint Louis, MO, USA). Sodium phosphate monobasic was purchased from Riedel-de Haën (Seelze, Germany). Sodium hydroxide pellets (Puriss meets the analytical specifications for BP, anhydrous, 98–100.5%) were purchased from Sigma-Aldrich (Saint Louis, MO, USA). Methanol and acetonitrile was purchased from Merck (Darmstadt, Germany).

### Determination of OLZ and DMO

In the literature various methods have been reported for quantification of OLZ and DMO such as employing HPLC coupled with atmospheric pressure chemical ionization mass spectrometry [17], MS/MS assay [18], [19], amperometric electrochemical detection with a solid-phase extraction procedure [20], [21], or coulometric detection [22], [23]. The HPLC-ECD apparatus available in our laboratory is a high sensitivity analytical cell containing two flow-through low volume working electrodes positioned serially, and is similar to the one used in Saracino’s study [24]. The chromatographic equipment included a Model 582 solvent delivery system coupled to a manual injector with a 20 µL fixed loop and an ESA (Chelmsford, USA) Coulochem III coulometric detector consisting of a Model 5020 guard cell and a Model 5010A improved standard analytical cell. The detection apparatus was similar to that used in Sabbioni’s study [25]. The apparatus was connected to a personal computer with SISC-32 chromatography data system software. The HPLC-ECD system such as a guard cell was set at +400 mV; the analytical cells were set at –200 mV (channel I) and +300 mV (channel II) [5]. In addition, the initial preliminary results using the mobile phase at near pH 7 also caused slight degrading of the silica gel in the YMC basic column [22], [26] and resulted in further shortening the useful life of analytical cells. Our laboratory therefore chose a cheaper Hypersil C18 column [27] and modified the mobile phase compositions and final pH to be 6.4.

Our preliminary results were carried out initially according to the methodology of Chiu’s study [26]. Since a used internal standard is not commercially available, triprolidine was initially used as IS while the pH of the mobile phase was adjusted to be 6 with an increasing percentage of phosphate buffer (Lin’s thesis; unpublished data). Under these conditions, a high working voltage (E1/E2 = +300 mV/+700 mV) was set to detect IS simultaneously with DMO/OLZ, and this resulted in an unstable background current in the analytical system [22] and large variation in detection. Because clozapine is seldom used for therapy in Taiwan due to its blood cell toxicity, this study used clozapine as the IS [25], [28] and working electrode (E2) voltage was set at +300 mV to compromise the overall sensitivity while E1, a reduction potential of –200 mV was chosen to minimize the possible background interference as much as possible [25].

The chromatographic separation was carried out on a Hypersil GOLD C18 column (150 mm ×4.6 mm ID). Before use, the mobile phase was filtered using 0.22 µm polyvinylidene dufluoride filters in a solvent filtration apparatus.

The solid-phase extraction procedure was according to Raggi’s study [20] except that we used a new strata C8 solid phase extraction cartridge (phenomenex) (30 mg, 1 mL). The cartridges were activated with 1 mL of methanol two times and conditioned with 1 mL of water five times. After loading the cartridge with 0.5 ml of patient plasma (or blank plasma) diluted with 0.5 mL of water and spiked with 80 ng/mL of the IS, the cartridge was washed with 1 mL of water two times and dried by applying full vacuum (ca. 40 kPa) for 2 min. The analytes were eluted from the cartridge with 1 mL of methanol, and full vacuum was again applied for 2 min.

### Assay validation

The method validation assays were performed according to the currently accepted U.S. FDA bioanalytical method validation guide [29]. Linearity was tested for the concentration rage of 1–100 ng/mL. In addition, blank samples were analyzed to exclude interferences. The acceptance criterion for the calibration curve was a standard deviation ≤20% at LLOQ and ≤15% for standards with a concentration above LLOQ. Furthermore, the correlation coefficient had to be no less than 0.999.

Intraday and the interday precision and accuracy were determined by assaying blank plasma spiked with at least 5 different known concentrations of both DMO and OLZ. Intraday precision was assessed by assaying spiked samples at each drug level (1, 5, 20, 40, and 100 ng/mL; n = 6). Precision was quantified by calculating intraday and interday coefficients of variation (CV%): [(SD/measured concentration)×100%]. Accuracy was expressed as percent error (%): [(measured concentration-spiked concentration)/spiked concentration]×100%. The recovery of N-desmethyl-olanzapine was determined at 5 different concentrations from 1 to 100 ng/mL.

OLZ and DMO in each sample were quantified by interpolation from linear calibration areas constructed by plotting peak area ratios (analyte to IS) as a function of the concentration of the calibrators. The calibrators, spiked with OLZ, DMO (1, 5, 20, 40, and 100 ng/mL) and clozapine 80 ng/mL, and a blank were obtained by the extraction procedure described above.

### Plasma samples and statistical analysis

The method was applied for a therapeutic drug monitoring study of nonsmoking patients (n = 48). All data were expressed as mean ± standard deviation. Statistical differences between 2 groups were evaluated by the unpaired Student’s t test following analysis of variance. As the different variables were not normally distributed, non-parametric statistical methods were used. The strength of the correlation between two parameters was analyzed by Spearman’s rank order correlation method (*r*_s_) using SigmaStat® 2.03. The modified Bonferroni’s method [30] was applied to adjust possible errors during multiple comparisons. A p value of less than 0.05 was considered statistically significant.

## Results

### Chromatography selectivity and assay validation

**Figure 1:**
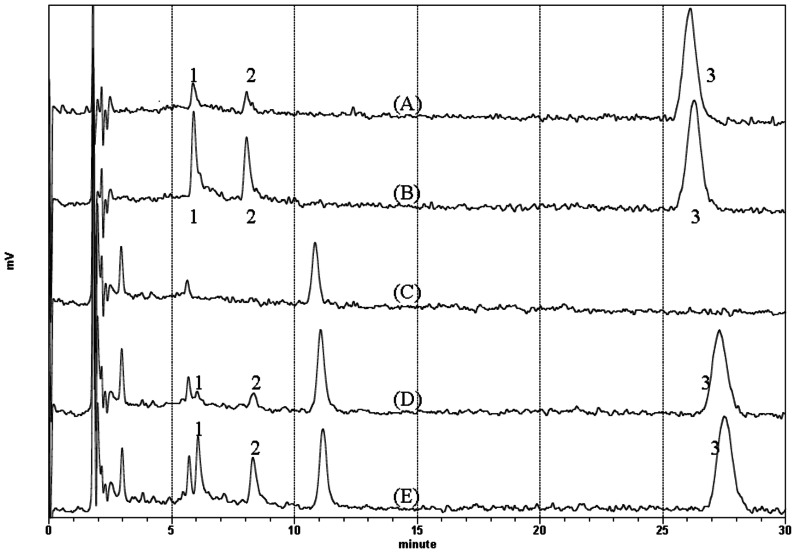
Representative chromatograms. (A) pure standard of 5 ng/ml N-desmethyl-olanzapine, 5 ng/ml olanzapine and 80 ng/ml clozapine dissolved in mobile phase without extraction; (B) pure standard of 20 ng/ml N-desmethyl-olanzapine, 20 ng/ml olanzapine and 80 ng/ml clozapine dissolved in mobile phase without extraction; (C) blank extracted plasma sample; (D) Extracted plasma sample spiked with 5 ng/ml N-desmethyl-olanzapine, 5 ng/ml olanzapine and 80 ng/ml clozapine; (E) Extracted plasma sample spiked with 20 ng/ml N-desmethyl-olanzapine, 20 ng/ml olanzapine and 80 ng/ml clozapine.

The run time of the assay was 30 minutes and the drugs were well resolved with retention times of 5.88 mins for DMO, 8.04 mins for OLZ and 26.27 mins for clozapine at a flow rate of 1 mL/min. The mobile phase consisted of 50 mM phosphate salt buffer (pH 5.7)-acetonitrile-methanol 67:22:11 v/v. The calibration curves were constructed using clozapine as the internal standard. The method exhibited good linear response for the concentration range of 1–100 ng/mL of each analyte. Representative chromatograms for standards of both analyates are shown in Figure 1 and the equation for the concentration of both analytes vs. ratio of peak area analyte/internal standard gave correlation coefficients equal to 0.999. LLOQ (the limitation of quantification) of each injection for DMO or OLZ was 0.02 ng (equivalent to 1 ng/mL). As shown in Table 1, results of the intraday and interday mean value accuracy for DMO or OLZ at tested concentrations were all within 15% of the actual value except at LLOQ, where it did not deviate by more than 20%. Results of the intraday and interday precision determined at each concentration level were all within 15% of the coefficient of variation (CV). The mean recoveries of DMO, OLZ from 1 to 100 ng/mL and that of IS were 86.22± 6.03%, 82.68± 6.53%, and 90.67%, respectively. The method was successfully used for the analysis of samples from patients treated with OLZ in the dose range of 5–20 mg/day.

**Table 1:** Precision and accuracy for the determination of olanzapine and N-desmethyl-olanzapine in spiked plasma.

Analyte	Spiked concentration (ng/mL)	Repeatability (CV %)	Accuracy (Error %)	Interday Precision (CV %)	Interday Accuracy (Error %)	Recovery (%)
olanzapine	1	19.76	–12.89	12.35	–12.17	73.26
	5	13.19	3.92	8.73	–8.91	88.08
	20	2.66	–11.65	3.99	–7.08	84.90
	40	2.50	7.81	2.56	8.22	84.38
	100	0.46	–1.08	3.87	–1.24	93.83
N-desmethyl-olanzpine	1	14.69	–4.04	6.95	–16.45	73.46
	5	8.31	11.76	13.95	–5.66	83.68
	20	8.64	–5.73	8.23	6.53	89.13
	40	3.27	7.92	3.08	5.83	78.31
	100	5.63	–3.86	5.08	–2.23	90.41

### Patient plasma samples and correlation of C/D ratios of OLZ or DMO level and patients biochemical parameters

**Figure 2:**
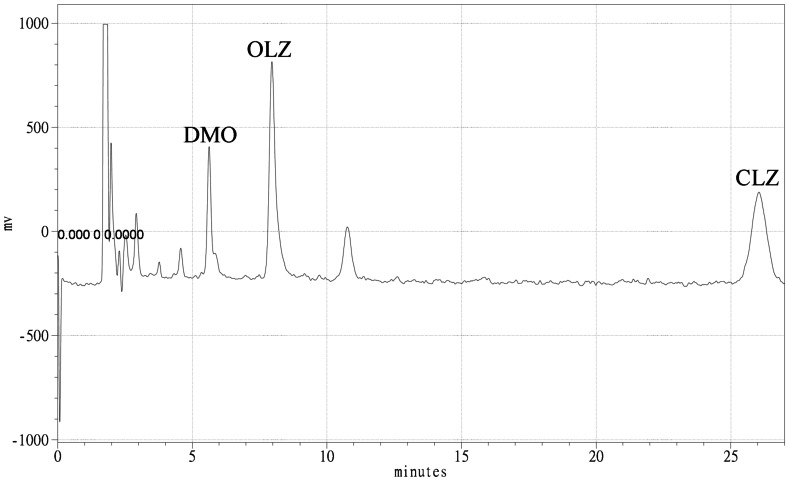
Chromatogram of a SPE extracted serum sample from a patient who received 20 mg of OLZ daily. DMO, N-Desmethyl-olanzapine; OLZ, olanzapine; CLZ, clozapine.

The chromatogram of a plasma sample from a patient who received 20 mg of OLZ daily is shown in Figure 2. The patient characteristics are shown in Table 2. The median levels of OLZ and DMO for all patients were 37.20 and 5.33 ng/mL, respectively. Among these patients, their plasma OLZ and DMO concentrations varied 42-fold (2.22 ng/mL – 93.98 ng/mL) and 16-fold (1.46 ng/mL – 22.79 ng/mL), respectively. The dose-corrected concentrations (C/D) varied more, 79-fold for OLZ and 23-fold for DMO. Results of median OLZ and DMO plasma C/D ratio were 2.41 and 0.51 ng/mL/mg, respectively. The median OLZ/DMO ratio was 4.40. The mean OLZ/DMO ratio was 6.85±6.42. OLZ level (*r*_s_ = 0.54, p<0.01) and OLZ normalized by body weight (*r*_s_ = 0.43, p<0.01) were positively correlated with the dose but, DMO (*r*_s_ = –0.42, p<0.01) and DMO normalized by body weight (*r*_s_ = –0.45, p<0.01) were negatively correlated with the escalation in dosage. Among female patients, the OLZ level (*r*_s_ = 0.59, p<0.01) and OLZ normalized by body weight (*r*_s_ = 0.52, p<0.01) were positively correlated with the dose. Among male patients, the OLZ level (*r*_s_ = 0.54, p<0.01) was positively correlated with the dose, but not OLZ normalized by body weight. Gender or age did not significantly affect OLZ/DMO drug levels or biochemical parameters.

**Table 2:** Summary of patients’ demographic and metabolic characteristics.

	Patients with Schizophrenia ‡		
	All (n = 48)	Female (n = 30)	Male (n = 18)
Age (y/o)	40.85±11.14	40.80±11.43	40.94±10.96
OLZ dose (mg)	14.43±5.24	14.25±5.30	14.72±5.28
Glucose (mg/dL)	93.00±15.70	92.63±17.81	93.61±11.83
Insulin (µU/mL)	11.61±6.13	12.33±6.17	10.40±6.03
Homocysteine (µmol/L)	11.88±4.63	11.26±3.83	12.90±5.71
C-peptide (ng/mL)	3.06±1.55	3.13±1.72	2.95±1.25
Prolactin (mg/dL)	24.94±15.52	27.45±17.71	20.74±10.05
Uric acid (mg/dL)	5.92±1.71	5.89±1.62	5.97±1.89
Cholesterol (mg/dL)	191.48±36.78	193.00±36.51	188.94±38.14
Triglyceride (mg/dL)	153.15±104.89	146.83±102.89	163.67±110.31
DMO levels (ng/mL)	7.03±4.50	6.80±3.62	7.42±5.77
OLZ levels (ng/mL)	35.46±24.12	41.65±26.16	25.15±16.15
DMO C/D (ng/mL/mg)	0.56±0.44	0.54±0.38	0.59±0.54
OLZ C/D (ng/mL/mg)	2.46±1.67	2.88±1.79	1.76±1.18
Ratio of OLZ/DMO	6.85±6.42	7.88±6.95	5.13±5.13

Data given as mean ± standard deviation.

‡ Patients were administered 5–20 mg olanzapine daily.

No significant differences between two genders of subjects.

DMO, N-Desmethyl-olanzapine; OLZ, olanzapine; C/D: concentration/dose.

Correlations of drug levels to various biochemical parameters were summarized in Table 3. Between biochemical parameters and the OLZ concentration or the OLZ C/D ratio no correlations were found. There were significant (p< 0.05) negative correlations between the DMO concentration with or without normalized by dose and the levels of glucose (*r*_s_ = – 0.42; *r*_s_ = – 0.45) and insulin (*r*_s_ = – 0.39; *r*_s_ = – 0.40), but the DMO concentration was positively correlated with homocysteine levels (*r*_s_ = +0.38). Moreover, after applying the modified Bonferroni’s method to adjust possible errors during multiple comparison, the correlations of DMO levels to glucose (adjusted p = 0.047), or DMO C/D ratios to insulin (adjusted p = 0.049) remain significant statistically, but the correlation between levels of DMO and homocysteine became marginally significant (adjusted p = 0.051).

**Table 3:** Correlations of olanzapine or desmethylolanzapine to individuals’ metabolic parameters in patients with schizophrenia (n = 48).

	DMO	DMO C/D	OLZ	OLZ C/D
Glucose				
*r_s_* =	–0.45†	–0.42	–0.08	–0.11
p =	0.00147	0.00335	0.609	0.469
Insulin				
*r_s_* =	–0.40	–0.39†	–0.16	–0.19
p =	0.00538	0.00608	0.283	0.191
c-peptide				
*r_s_* =	–0.22	–0.27	0.06	–0.06
p =	0.128	0.0593	0.677	0.701
Homocysteine				
*r_s_* =	+0.38	0.28	0.04	–0.04
p =	0.00801	0.0587	0.786	0.801
Prolactin				
*r_s_* =	0.08	0.11	0.09	0.10
p =	0.591	0.451	0.551	0.504
Uric acid				
*r_s_* =	–0.09	–0.20	0.0331	0.0285
p =	0.545	0.177	0.822	0.846
Cholesterol				
*r_s_* =	0.04	–0.02	0.01	0.10
p =	0.773	0.889	0.934	0.485
Triglyceride				
*r_s_* =	–0.10	–0.19	–0.07	–0.07
p =	0.474	0.192	0.621	0.624

Plasma olanzapine or desmethylolanzapine concentrations corrected by individual’s dosage were expressed as OLZ C/D or DMO C/D.

Spearman’s correlation coefficients were expressed as *r_s_* and p values were uncorrected for multiple comparisons.

† Correlations remain statistically significant following post hoc modified Bonferroni correction (adjusted p value <0.05).

## Discussion

OLZ is one of the most prescribed antipsychotic drugs for the treatment of schizophrenia and bipolar disorder. The results of this study demonstrated significant interindividual variation in OLZ and DMO concentrations among patients with schizophrenia. These data are in accordance with several previous studies [4], [5] Due to the wide interindividual variability in the pharmacokinetic properties of OLZ, therapeutic drug monitoring (TDM) became a valuable tool for the guidance of psychopharmacotherapy [5]. The TDM group of the Arbeitsgemeinschaft für Neuropsychopharmakologie und Pharmakopsychiatrie (AGNP) issued best practice guidelines for TDM in psychiatry in 2004 [31] and updated the guidelines in 2011 [32]. TDM for OLZ is strongly recommended (Level 1 recommendation). A Level 1 recommendation is defined as one that clinical studies have shown to have beneficial effects of TDM on therapeutic efficacy and tolerance. The recommended drug concentration and laboratory alert level for OLZ by AGNP-TDM guidelines is 20–80 ng/mL and 150 ng/mL, respectively [32]; however, a correlation of OLZ concentrations with efficacy and adverse reactions has not been well established in the literature [33].

Results of validation assays for the current analytical methodology were satisfactory. Compared to LC/MS/MS, used HPLC-ECD analytical apparatus was less expensive, its maintenance was easier, and waste production and expenses for organic solvents were also much less. This established HPLC-ECD system is quite stable and easily maintained for sample analysis without using the pre-column as compared to Saracino’s methods [24]. When compared to the other coulometric studies, our LLOQ for OLZ or DMO was 0.02 ng (equivalent to 1 ng/mL) which was an improvement compared to that (0.1 ng OLZ) of Chiu’s study and that (15 nmol/L equivalent to 1.56 ng/mL) of Skogh’s study [5] even though it was not as good as Saracino’s study (0.1 ng/mL). Due to the late retention time for IS (CLZ; 26.3 min), however, each complete sample analysis required 30 mins.

The strata C8 SPE column was used in this study because of more economical, simpler procedures and better (non-inferior) recovery than the others (Varian LRC certify I [22]; Isolute HCX [5]; Bond Elute LRC [26]; diol sorbent [25]; BondElute phenyl [23]). Our overall extraction percentages for DMO, OLZ, clozapine were 87.04+3.8%, 92.95+3.60%, 88.27+2.88%, respectively, which is an improvement when compared to the extraction method for patients’ plasma used in Skogh’s study [5], although recovery of analytes was not as good as in Saracino’s study [23](> 90% for all analytes).

Accumulating clinical evidence indicates that OLZ harbors an increased risk of metabolic disturbances, including obesity, diabetes, dyslipidemia, and metabolic syndrome [34]. The underlying mechanisms of metabolic abnormalities are likely to be multifactorial and peripheral glucoregulatory dysfunction probably plays an important role [35]. OLZ might have a direct effect on insulin secretion or insulin action [36], [37]. In this study, the levels of metabolic parameters did not correlate with the OLZ concentration. Instead, the levels of glucose and insulin correlated inversely with the DMO concentration. These findings, which are in accord with previous study results [8], [14], revealed that DMO seemed to have a neutralizing effect on OLZ-induced metabolic changes. The probable concentration-dependent metabolic adverse reactions of OLZ might be complicated by the counteracting effects of DMO [13].

To the best of our knowledge, this is the first study examining the association between levels of DMO and homocysteine. Homocysteine is a risk factor for cardiovascular disease and is also associated with several neurological and psychiatric diseases [38]. However, data about the level of homocysteine in schizophrenia is conflicting and contradictory [39]. Some authors [40] have reported no changes, but others [41], [42] have reported increased levels, in untreated patients with schizophrenia. It has also been reported that patients with the 677TT genotype showed higher homocysteine levels than other patients and it was suggested that supplemental folate may be beneficial to some patients with schizophrenia and homocysteinemia due to the genetic defect of methylenetetrahydrofolate reductase [43], [44].

The mean homocysteine level for the currently recruited patients with schizophrenia was very similar to the results of two previous studies [15], [45]. Glucose and insulin seem to influence the metabolism of homocysteine, possibly through affecting the activity of key enzymes in homocysteine metabolism, including methylenetetrahydrofolate reductase and cystathionine-β-synthase [46]. Patients with schizophrenia and impaired fasting glucose had higher homocysteine levels than those with normal fasting glucose [47]; however, it is noteworthy that, in our study, the mean values of serum homocysteine in both genders were lower than 15 µmol/L, which is a cut-off point for hyperhomocysteinaemia, and only 8/48 patients had an abnormal serum homocysteine. Moreover, after applying the modified Bonferroni’s method to adjust possible errors during multiple comparisons, there is marginally significant correlation between levels of DMO and homocysteine (adjusted p = 0.051). Further investigation is needed to confirm such marginal results.

The generalization of the findings of our study should be cautious because it had some limitations. First, the sample size of our study was small and all subjects were non-smokers. Second, genetic variation in metabolic enzymes for OLZ (e.g., UGT1A4 and CYP1A2) was not analyzed. However, the influence of functional polymorphism for OLZ metabolic enzymes on plasma OLZ concentrations remained unclear. With respect to UGT1A4, the 142T>G single nucleotide polymorphism (SNP) was found to decrease plasma OLZ concentrations [48] or have no effect [49]. With respect to CYP1A2, the functional SNP of CYP1A2 was reported to have a significant impact on plasma OLZ concentrations [50] or did not affect OLZ plasma levels [49].

In conclusion, this study supported the reported negative correlation between glucose/insulin and DMO levels; however, in some cases, the probable normalized effects of DMO levels may not be able to correct the observed hyperhomocysteinemia which is considered to be a characteristic metabolic abnormality in several pathological conditions, including hypertension, diabetes and alcoholic liver disease. The underlying mechanisms for DMO effects on the observed metabolic changes and adverse cardiovascular activity require further investigation.
